# Erratum: Fifteen-Year Results of Cementless Medial Oxford Unicompartmental Knee Arthroplasty: Reliable Long-Term Fixation

**DOI:** 10.2106/JBJS.OA.ER.26.00140

**Published:** 2026-08-27

**Authors:** Nisrina Widari, Cathy Jenkins, Christopher Dodd, Stephen Mellon, David Murray

In the article by Widari et al., appearing in *JBJS Open Access*, Vol. 11, No. 2, e26.00140, entitled “Fifteen-Year Results of Cementless Medial Oxford Unicompartmental Knee Arthroplasty: Reliable Long-Term Fixation,” there were errors.

Specifically, Figures 1 and 2 were had errors and needed to be replaced with the below images.

**Fig. 1:**
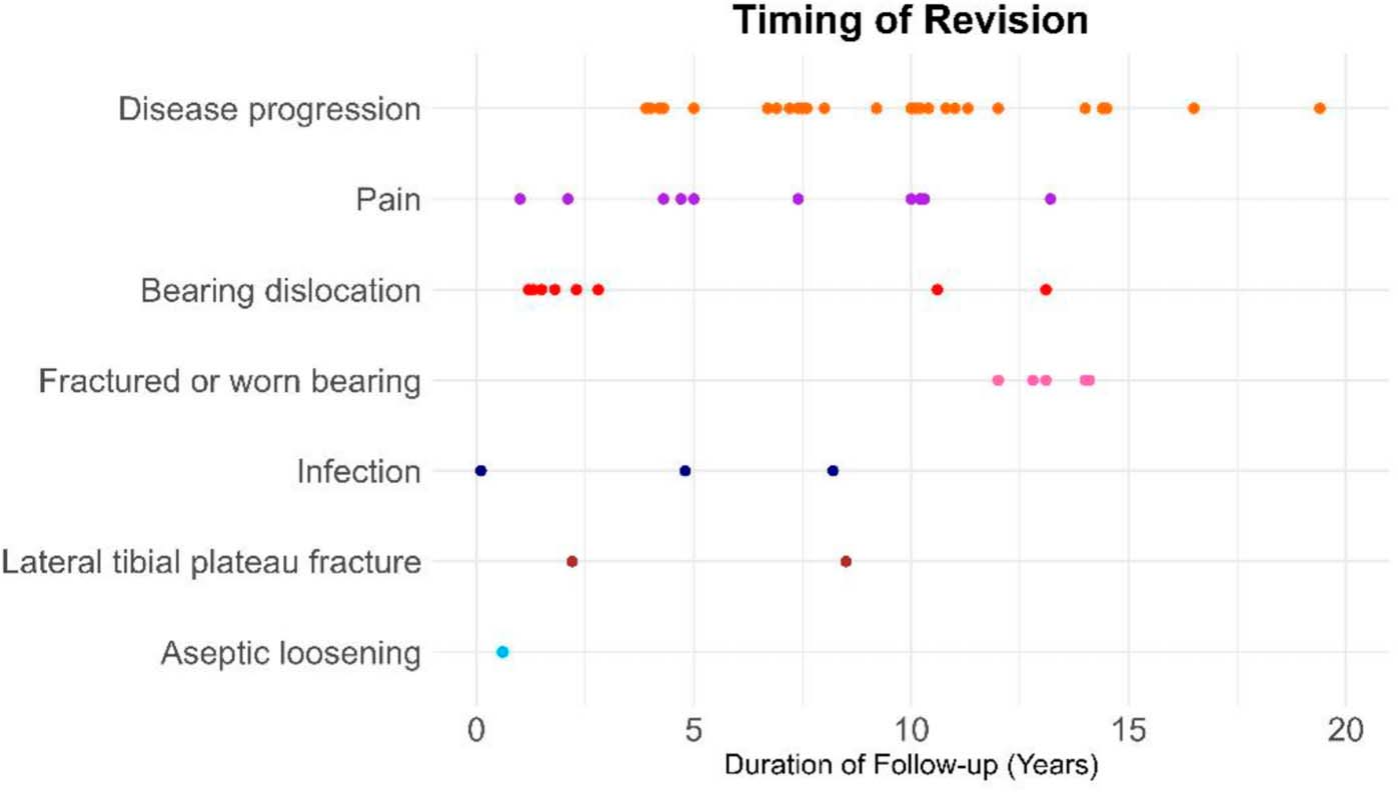


**Fig. 2:**
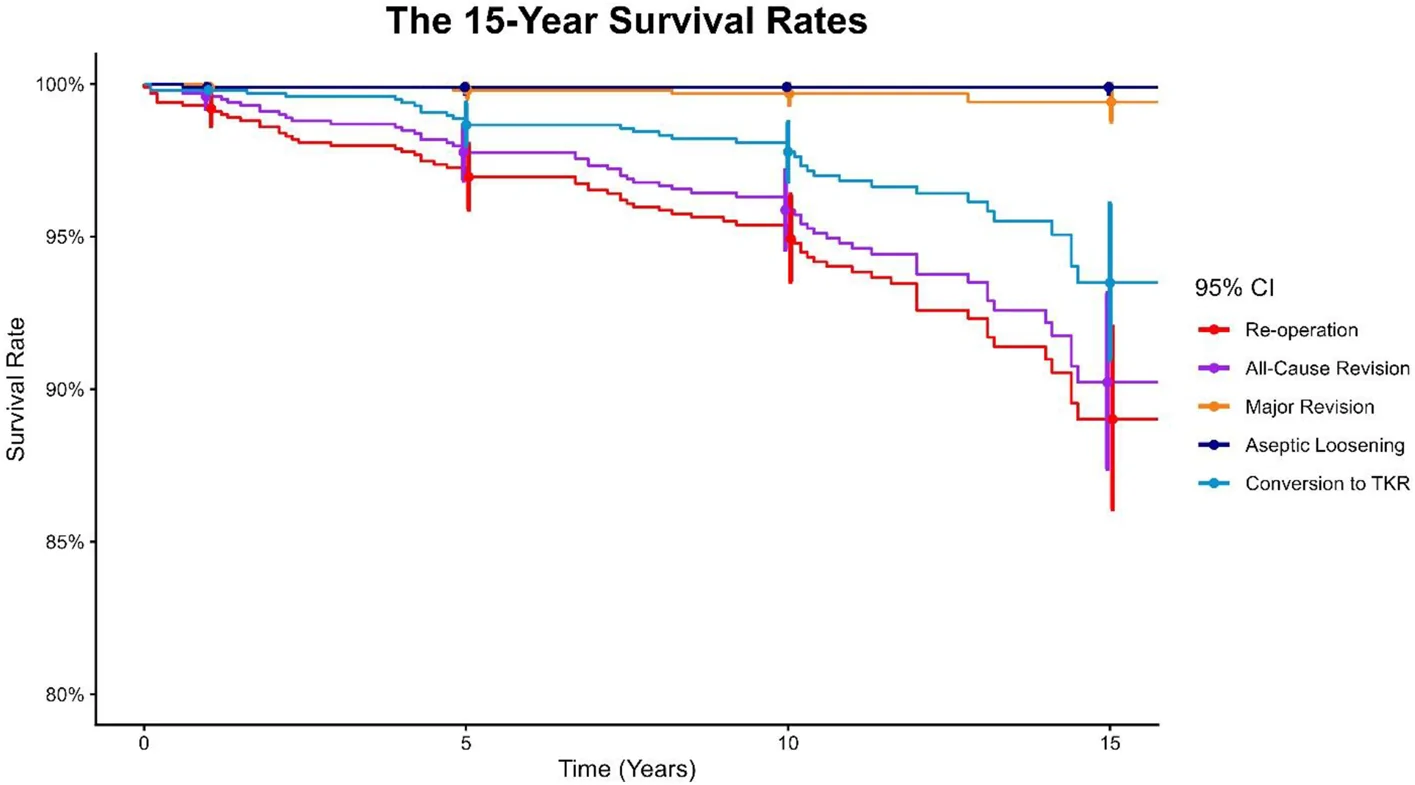


The errors have been corrected in the article online. The authors regret their oversight.
